# Experimental Study on the Cutting Process of Single Triticale Straws

**DOI:** 10.3390/ma16113943

**Published:** 2023-05-24

**Authors:** Dominik Wilczyński, Krzysztof Talaśka, Krzysztof Wałęsa, Dominik Wojtkowiak, Michał Bembenek

**Affiliations:** 1 Faculty of Mechanical Engineering, Institute of Machine Design, Poznan University of Technology, Piotrowo Str. 3, 60-965 Poznań, Poland; dominik.wilczynski@put.poznan.pl (D.W.); krzysztof.talaska@put.poznan.pl (K.T.); krzysztof.walesa@put.poznan.pl (K.W.); dominik.wojtkowiak@put.poznan.pl (D.W.); 2 Department of Manufacturing Systems, Faculty of Mechanical Engineering and Robotics, AGH University of Science and Technology, Mickiewicza Ave. 30, 30-059 Kraków, Poland

**Keywords:** biomass, straw-cutting process, cutting parameters

## Abstract

This paper presents experimental research on cutting a single stalk of triticale straw for the production of biofuel in the process of its compaction using the piston technique. In the first stage of the experimental study of cutting single triticale straws, the variable parameters were the moisture contents of the stem equal to 10% and 40%, the offset between the blade and the counter-blade *g*, and the linear velocity of the knife blade *V*. The blade angle and rake angle were equal to *α* = 0° and *β* = 0°. In the second stage, the variables, including the blade angle values *α* = 0°, 15°, 30°, and 45° and the rake angle values *β* = 5°, 15°, and 30°, were introduced. Taking into account the analysis of the distribution of forces on the knife edge leading to the determination of the force quotients *F_c_*″/*F_c_* and *F_w_*/*F_c_*, and on the basis of the optimization performed and the adopted optimization criteria, the optimal knife edge angle *α* can be determined (at values *g* = 0.1 mm and *V* = 8 mm/s) at *α* ≅ 0° and the angle of attack *β* within the range of 5–26°. What the value will be in this range depends on the value of the weight adopted in the optimization. The choice of their values may be decided by the constructor of the cutting device.

## 1. Introduction

The two main sources of biomass for energy generation are energy crops and waste [1]. Energy crops, such as silvergrass and woody plants, are grown primarily for the purpose of generating energy. The utilization of agricultural waste, e.g., barley, canola, oat, and wheat straw, for energy generation allows for increasing the value of existing crops [2]. In reality, such waste products constitute a plentiful, cheap, and easily available source of renewable lignocellulosic biomass [3]. It is assumed that biomass constitutes an alternative source of energy that, after processing to achieve specific physical and chemical characteristics, may replace fossil fuels to a significant degree. An important strategic goal of the Member States of the European Union has been to systematically increase the use of biomass in energy generation [4,5,6]. The primary issue associated with agricultural straw is the relatively low density of the material, which is problematic from the standpoint of its transportation and storage [7]. The compaction of loose biomass allows for improving its energy characteristics. Recently, with the increase in the utilization of biofuel as an energy source, there has been rapid development of the machinery and devices necessary to carry out this process. In order to design a device that allows for obtaining biofuel with good energy characteristics, one needs to examine the characteristics of the briquette in order to determine the variables for the compaction process as well as the degree of fragmentation of the biomass for compaction [8,9,10,11,12,13,14,15].

Therefore, biomass cutting and fragmentation is an important process stage in the production of biofuel and food. Important considerations include the energy efficiency of the process and achieving the desired biomass particle size, which affects the physical, chemical, and mechanical properties of the resulting biofuel [16,17,18,19,20,21].

There are several biological and structural characteristics of cellulosic biomass that affect the energy required for its fragmentation (cutting). Lignocellulose plant materials exhibit orthotropic mechanical characteristics, which vary depending on the orientation of the particles relative to the direction of the strands in the material [19].

Numerous researchers from all over the world have taken an interest in the issues related to the cutting and fragmentation of biomass, such as the geometry of the cutting blades, the kinematics of the cutting device, and the moisture content of the cut biomass, together with the degree of its deformation.

Bitra et al., (2009) examined the shredding process for, i.e., wheat straw for different sieve aperture dimensions of the knife mill, rotation, and feeding speed. The total specific energy of the fragmentation process increased with the rotation speed of the knife mill. On the other hand, the energy decreased with an increase in the sieve aperture size. Its value was lower with an increase in the biomass feeding speed [22].

Zastempowski and Bochat (2020) carried out an examination of the cutting process of rye straw into sections of specific lengths using a cutting drum of classical and novel design. The classical design of the cutting drum was cylindrical for transverse cutting of the material. In contrast, the novel design of the cutting drum was based on a double truncated cone. As a result, the cutting action made diagonal cuts in two directions. Therefore, the examination was carried out using four cutting drum designs with the cutting angles *α* = 0°, 15°, 30°, and 45°. The study evaluated the efficiency of the cutting process and the per-unit energy consumption together with the cutting resistance per unit. The novel design of the cutting drum increased the efficiency of the process by up to 25%, reducing the per-unit energy consumption by up to 34%. On the other hand, the cutting resistance per unit was lowered by 8% [23].

Others have searched for the influence of the moisture content of the cut material and its chemical composition, e.g., wheat straw, rice straw, corn stalks, and flowers, as well as sorghum and miscanthus. It has generally been found that reducing the moisture content of the cut material reduces the energy intensity of the process. For example, Abdellatif Barakat et al., as a result of reducing the initial moisture content of the straw from 7% to 1%, obtained a decrease in the energy consumption of the process during the grinding of wheat straw from an initial straw size of 300–600 mm to a particle size below 0.25 mm. In turn, the lowest cutting force of triticale straw was obtained for the lower moisture content, which was 16.94%, during cutting with a knife drum, where the blade of the knife had a helical geometry, the blade knife angle was 45°, and the rake angle was 15° [24,25,26].

Numerous researchers are studying the cutting process of biomass materials of different origins. Gao et al., (2022) examined the process of the supported cutting of Caragana korshinskii stems. In the course of the experiment, an increase in the cutting force increased the specific energy of the cutting process and the stem diameter, whereas the above parameters decreased with an increase in the moisture content of the cut material. The use of knife blades with angle values in the range of 20–35° resulted in increasing the cutting force, with a tendency to reduce the value of the specific energy. When utilizing knife blades with angle values in the range of 0–20°, the force and energy values were lower. The authors of the cited work carried out an optimization by applying the multi-parameter Box–Behnken test. The determined optimal values were 0.5 m/s for the linear velocity of the blade, 25° for the blade angle, 20° for the rake angle, and 1.4 mm for the offset between the blade and the counter-blade [27].

Zhang et al., (2019), in a study of cutting rice stems, demonstrated that selecting an appropriate angle for blade sharpening and the height of the cutting point had a significant effect on energy consumption. The maximum cutting force decreased together with the sharpening angle of the blade. For cutting without the counter-blade (unsupported cutting), the optimal sharpening angle of the blade was 45°, whereas for cutting with the counter-blade (supported cutting), the optimal angle was 30°. The researchers used the following blade angles in the study: 0°, 30°, 45°, and 60° [28].

An examination of the cutting process of sisal leaves by Song et al., (2022) led to the conclusion that process parameters have a material bearing on the shear stress and specific energy of the cut. It was determined that at higher cutting speeds, the shear stress and cutting energy could be reduced. The cited researchers achieved a minimum value of the shear stress and specific energy for the cutting process for a blade angle equal to 40° and a rake angle of 45°, with knife blade velocity rates of 5 and 3 m/s, respectively [29].

Van-Dam Vu et al., (2020) studied the cutting process of corn stalks and determined that the proper selection of the process parameters allows for reducing the cutting force and energy by a factor of 2, 3, and up to 4. They observed that at a higher linear velocity of the blade, there is high energy consumption with a low-value cutting force [30].

The placement of the cutting point has a material influence on the energy consumption of the canola stem-cutting process, as concluded by Mohsen Azadbakht et al. following an examination of this type of biomass considering different values of moisture content [25].

Sunil K. Mathanker et al. concluded that the correct selection of the process parameters, i.e., the cutting speed and the angle of the blade, reduced the energy consumption of the cutting process of energy cane, and the value of specific energy of the cut was correlated with the variable cross-section area of the stem [31].

An examination of the cutting process of silvergrass by Phillip C. Johnson et al. led to the conclusion that optimizing the cutting speed and the blade angle had a positive effect on energy consumption and increased the efficiency of the machine. The cutting energy was directly proportional to the knife blade velocity and stem diameter [32].

In the studies cited above, the researchers analyzed the effect of the blade angle, the rake angle of the cutting blade, its speed, the moisture content of the material being cut, its geometric parameters related to the cross-section at the point of the cut on the energy, and the power required to sever the biomass material with specific physical and mechanical characteristics.

Biomass that has been fragmented to the specific particle size undergoes processes such as agglomeration in order to produce biofuel. One can therefore assert that the energy efficiency of the cutting process is a component of the energy efficiency of the biofuel manufacturing process and, therefore, significantly affects the viability of its generation, in addition to the aspect of sustainability, which is of primary concern.

The above considerations were the basis for the study presented in this work, which examined the cutting process of the Belcanto variety of triticale straw utilizing the testing station based on the MTS Insight 50kN testing machine (manufacturer: MTS Company, USA). The material used in the study is easily available in the region where the authors’ research unit is located. The experimental study and its results are to be used for the construction of a highly efficient device for cutting triticale straw for the purpose of its compaction and obtaining biofuel.

## 2. Materials and Methods

### Material Preparation

The straw, which was harvested from the fields located in the Wielkopolska Province (west-central Poland) at the geographical coordinates 52°00′08.0″ N 17°46′46.9″ E, was used as a testing material to conduct the presented research. The straw was gathered by hand during the harvest season. The plants were harvested just above the soil surface, in the immediate vicinity of the root. The harvest took place in the final stage of the plants’ life cycle—99 on the BBCH scale, which stands for full maturity and the end of the second stage of dormancy. The straw underwent seasoning for a period of twelve months after harvesting, contained indoors at room temperature. The straw moisture content was tested with a Mettler Toledo analyzer (manufacturer: Mettler-Toledo International Inc., Zurich, Switzerland). The average value from ten measurements was 10 ± 0.48%. The experimental study was carried out in two stages. The first stage focused on the examination of the knife with a blade angle of *α* = 0° (it should be understood that the knife was unsharpened, applicable to both stages of the study). In this way, the authors of the study wanted to achieve the conditions approximating pure shear to the furthest possible degree in order to determine the influence of blade velocity, the offset between the blade and the counter-blade, as well as the moisture content in the material on the value of the cutting force *F_c_*. ANOVA variance analysis was carried out for the interactions among these input variables of the process (process set-points), enabling the determination of the parameter values at which the value of the cutting force was the lowest. These parameters (set-points), i.e., the moisture content of the material equal to 10%, the offset between the blade and the counter-blade *g* = 0.1 mm, and the knife velocity *V* = 8 mm/s, were afterward used as constants in the second stage of the study. Subsequently, the focus of the study in its second stage was to observe and evaluate the effect of the changes in the values of the blade angles *α* = 0°, 15°, 30°, and 45° as well as the rake angles *β* = 5°, 15°, and 30°. For the above input parameters of the cutting process, in the second stage of the study, a multi-criteria ANOVA analysis was also carried out, indicating which values of the blade angle *α* and the rake angle *β* the cutting force achieved the lowest value. The authors suggested an optimization for selecting the cutting process parameters, employing the criteria of the ratio of the horizontal component of the cutting force *F_c_* to the vertical component force (measured experimentally) *F_c_*″/*F_c_* as well as the cutting process efficiency criterion, which was defined by the authors and expressed as the ratio *F_w_*/*F_c_*.

**Figure 1:**
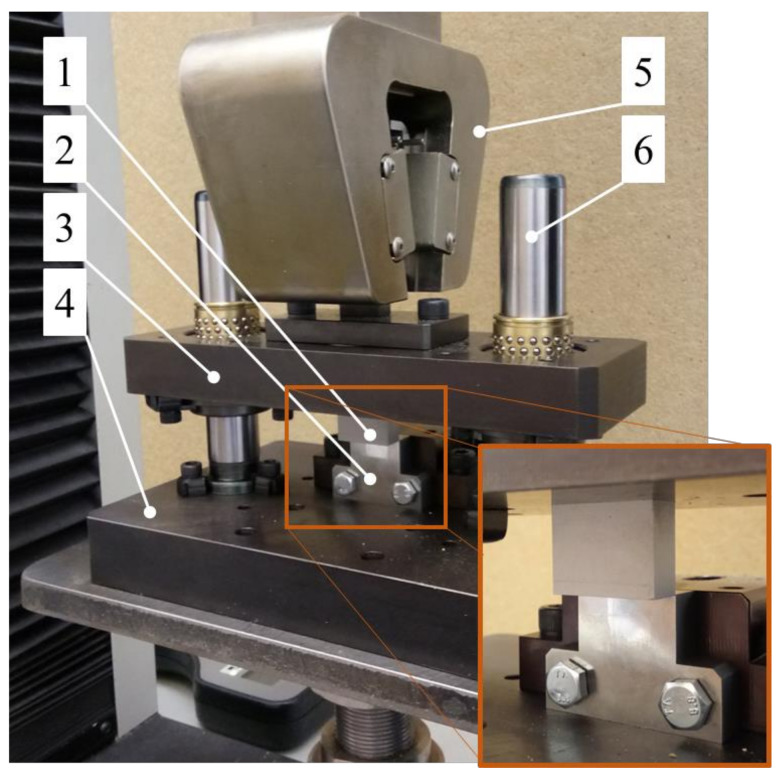
Testing station installed on testing machine: 1—cutting blade, 2—counter-blade, 3—movable upper plate, 4—fixed lower plate, 5—movable clamp of the testing machine, 6—guide with bearings.

**Figure 2:**
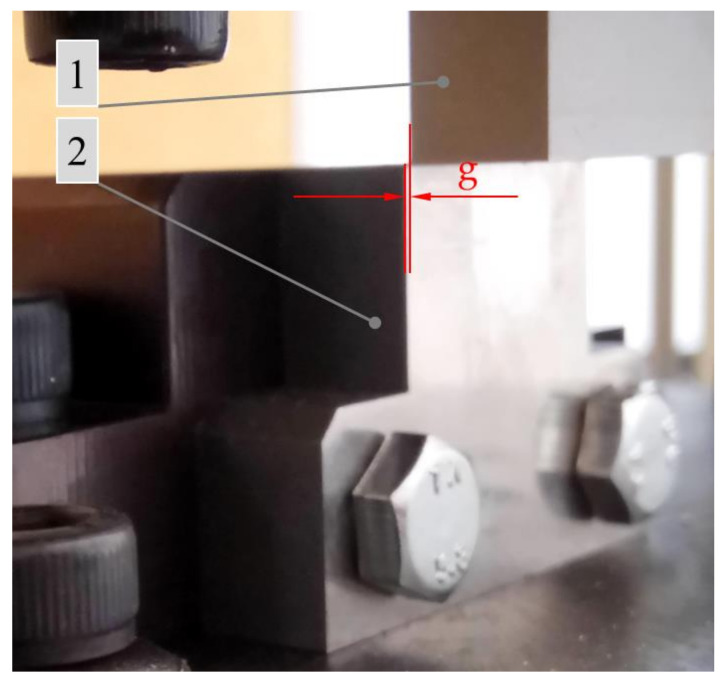
General view illustrating the offset *g* between the movable blade of the knife and the stationary counter-blade, where 1—cutting knife, 2—counter-blade.

The testing station used in the first stage of the study of cutting the straw is presented in Figure 1. The examination entailed an attempt at the simple shearing of singular straw stems on the testing machines with an installed cutting station (Figure 1) in order to determine the value of the cutting force *F_c_* for the different values of the cutting velocity *V* (velocity of the cutting edge of the blade), the spacing width *g* (see Figure 2) between the blade and the counter-blade, and two values of moisture content of the straw. The cutting force *F_c_* was recorded by the sensor of the MTS Insight 50 kN testing machine (manufacturer: MTS Company, Eden Prairie, MN, USA). The shear of the stem was performed at a height approximately 50 mm from the end after cutting the stem above ground level during harvest. The cross-section of the stem, at this point, had the largest area. Therefore, the highest values of the shearing force were obtained relative to the cutting points located higher up the stem, which were characterized by the smaller dimensions of the cross-section (the same principle for selecting the cutting point was assumed in the second stage of the examination). Cereal stem is a natural material, and therefore, it is difficult to ensure identical dimensions and mechanical characteristics of different plant stems. In order to obtain comparable results in the study, the samples were selected from stems with a similar external diameter at the planned cutting point (the external stem diameter was between 5 mm and 5.5 mm) as well as with similar side thickness. Before measurement, the knife blade was positioned approximately 2 mm away from the stem surface.

The testing station used in the test (see Figure 1) comprised two plates. The movable upper plate (3) was affixed to the movable clamp (5) of the testing machine. The plate (3) executed a reciprocating motion effected by the movement of the clamp (5) of the testing machine. The motion was carried out relative to the fixed lower plate (4) of the testing station thanks to two guides with bearings (6) affixed to the lower plate (4). The cutting knife (1) with a blade angle *α* = 0° and a rake angle *β* = 0° was affixed to the movable plate (3). The counter-blade (2) was affixed to the lower plate (4) (see Figure 1). The variable parameters in the first stage of the experimental study were: the offset *g* between the blade of the knife (1) and the counter-blade (2) was equal to *g* = 0.05, 0.1, and 0.2 mm (see Figure 2), and the blade velocity *V* was equal to *V* = 0.5, 1, 2, 4, and 8 mm/s. For each value of the offset *g,* the effects of all the blade velocity values *V* were examined. For each set of parameters, the test was repeated ten times.

**Figure 3:**
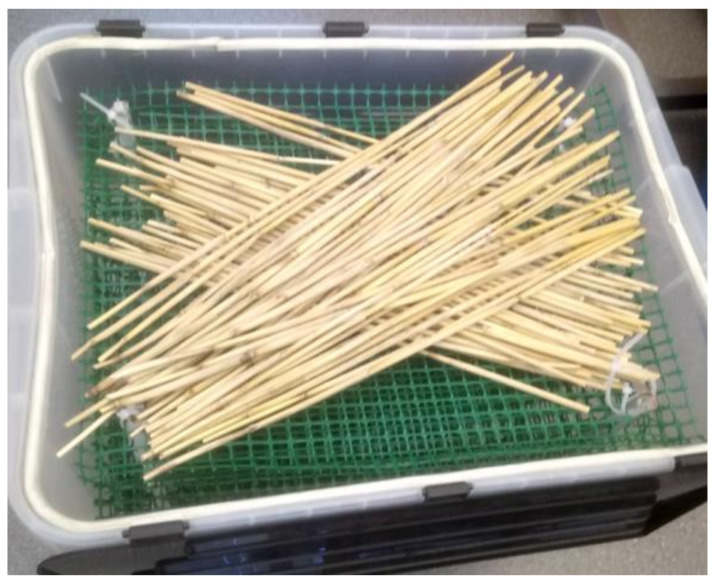
General view of a container for seasoning the straw to achieve a higher moisture content in the material.

The testing was carried out for two levels of stem moisture content. As before, dry straw with a moisture content of 10% and moist straw was kept in special lockable containers to which warm water was introduced to evaporate and moisturize the straw (see Figure 3) in order to achieve the required level of moisture content. Approximately 1–2 L of hot water was poured into the bottom of the container, and the container was closed. The straw moisture was measured daily until it reached 40%.

The moisture content in the seasoned straw samples was examined using the earlier-described scale dryer. The average value of ten measurements was 40 ± 1.14%.

**Figure 4:**
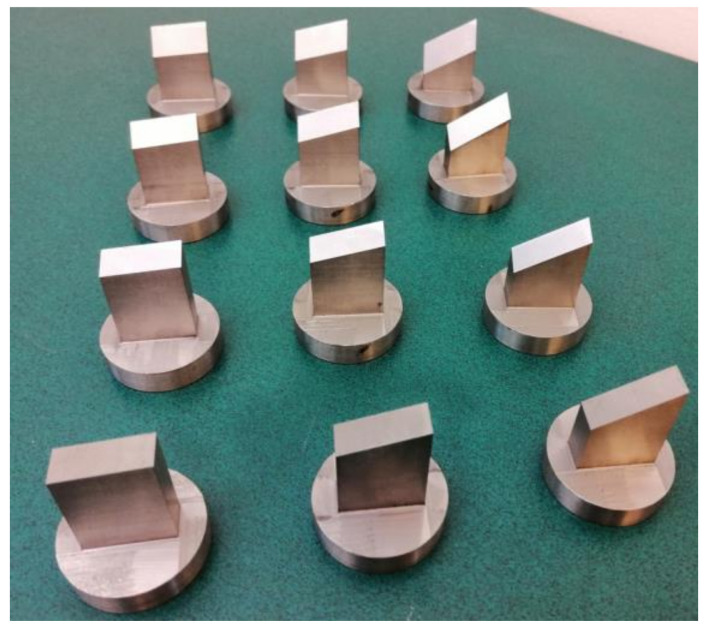
General view of the knives used in the second stage of the experimental study of the cutting process of straw stems.

The second stage entailed examining the cutting process of the straw stem with different values of the blade angle, *α* = 0°, 15°, 30°, and 45°, blade rake angles *β* = 5°, 15°, and 30°, with the offset *g* = 0.1 mm, linear velocity of the blade equal to *V* = 8 mm/s, and moisture content in the straw stems of 10%, as the two latter parameters led to achieving the lowest values of the cutting force *F_c_* in the first stage of the study. In summary, the 12 knives presented in Figure 4 were employed in the second stage of the examination.

**Figure 5:**
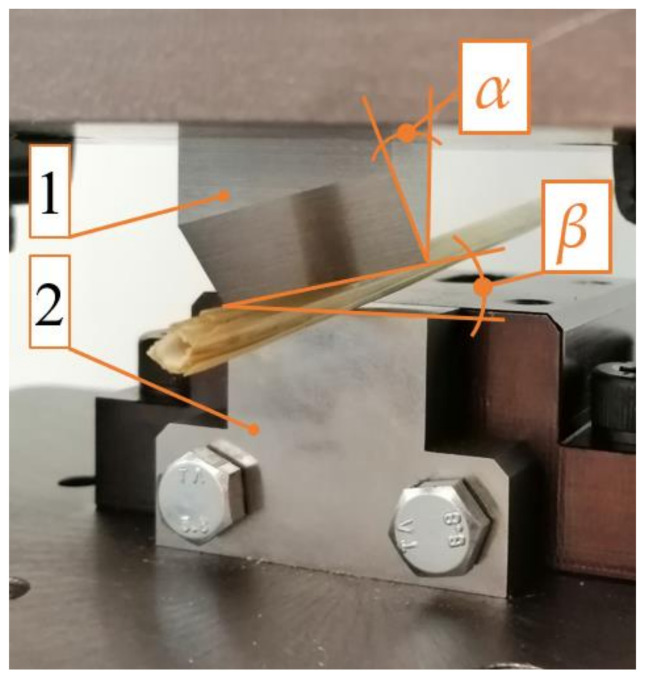
The testing station for the cutting process of straw stems utilized in the second stage of the study: 1—knife with blade angle *α* > 0° and *β* > 0°, 2—counter-blade.

The examination carried out in the second stage of the study may be considered a continuation of the examination described earlier. It was carried out on the same testing station installed on the MTS Insight 50 kN testing machine (manufacturer: MTS Company, Eden Prairiem, MN, USA) (see Figure 1) with installed knives (1) with variable blade angles *α* and rake angles *β* (see Figure 5). The testing station was equipped with a counter-blade (2) (see Figure 1), which could be moved linearly in the horizontal direction for the purpose of setting the offset *g* spacing between the blade (1), and the counter-blade (2) (see Figure 2). In the second stage of the study, this value was equal to *g* = 0.1 mm. The aim of this examination was to determine the influence of the variance in the angle values *α* and *β* on the value of the cutting force *F_c_* (see Figure 5).

## 3. Results

**Figure 6:**
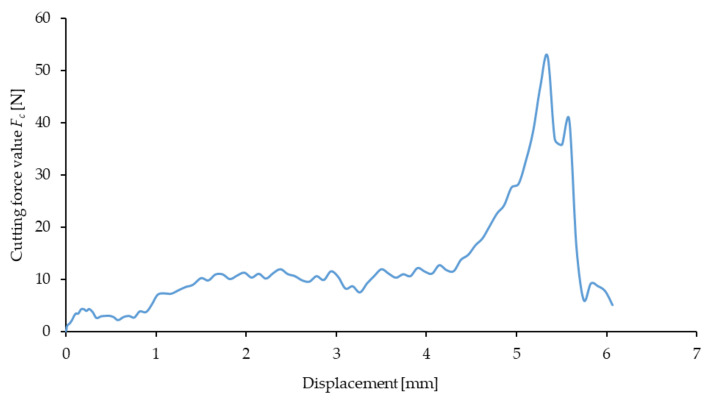
An example characteristic curve of variance of the cutting force *F_c_* for a *g* value of 0.1 mm and blade velocity *V* = 8 mm/s in the course of the cutting process for straw with a moisture content of 10%.

Figure 6 demonstrates an example characteristic curve describing the variance of the cutting force as a function of knife blade displacement.

### 3.1. The Results of the Examination Carried Out in the First Stage of the Study for Blade Angle α = 0° and Rake Angle β = 0°

Table 1 shows a breakdown of the average values of the cutting force measured for all the tests carried out for the straw with moisture contents of both 10% and 40%.

The results obtained gave grounds for performing a variance analysis of the input parameters of the experiment, with the response being the cutting force value *F_c_* measured by the testing machine.

#### 3.1.1. Multivariate Analysis of Cutting Force F_c_ for Cutting Straw with a Moisture Content of 10%

**Figure 7:**
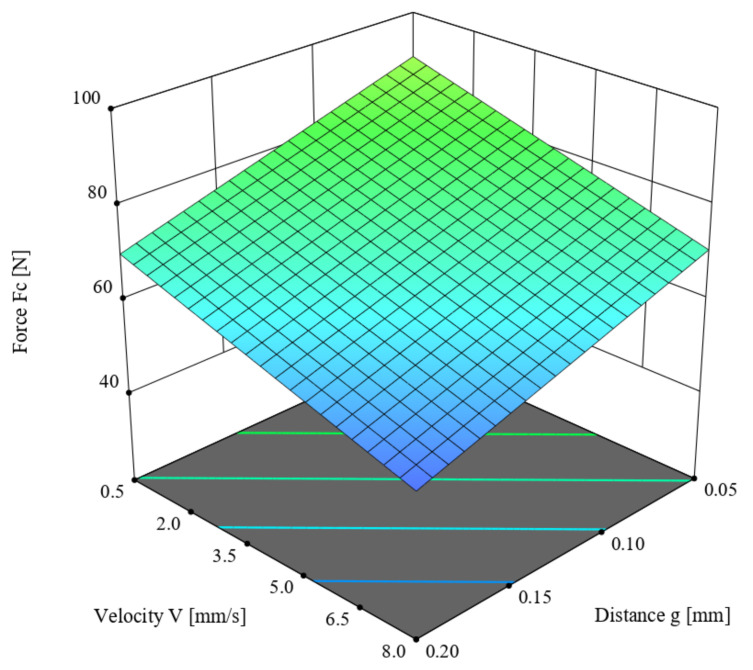
The variance of the cutting force *F_c_* as a function of the offset *g* between the blade and the counter-blade, as well as the linear velocity of the blade *V* and moisture content of 10%.

This chapter describes the ANOVA variance analysis performed on the value of the cutting force *F_c_* (N) for the straw stem with a moisture content of 10%, depending on the offset between the blade and the counter-blade *g* (mm) as well as the knife blade velocity *V* (mm/s). Table 2 presents obtained results. The linear model was employed in the analysis based on the criterion of statistical significance *p* = 0.1, for which R^2^ = 0.3581. According to Table 2, the F-value of the model was 33.5, which means the model was significant. Meanwhile, the *p*-value for individual model components was lower than 0.05, which confirms the significance of these components. The difference between the predicted R^2^ = 0.1117 and the adjusted R^2^ = 0.2511 coefficients of determination, which was lower than 0.2, along with the Adeq Precision value of 5.3969, indicates the usefulness of the model to estimate the value of the force. The obtained model is presented in Formula (1), and its graphical representation is shown in Figure 7.

*F_c_* = 98.20783 − 133.61173 × *g* − 2.59060 × *V*, (1)

Here, *F_c_* is the cutting force (N), *g* is the offset between the blade and the counter-blade (mm), and *V* is the knife blade velocity (mm/s)

In analyzing the values of the parameters in Table 2, both input variables for the straw-cutting process had a significant influence on the value of the cutting force *F_c_*. The lowest value of the cutting force *F_c_* was achieved for the offset *g* = 0.2 mm and velocity *V* = 8 mm/s.

#### 3.1.2. Multivariate Analysis of Cutting Force F_c_ for Cutting Straw with a Moisture Content of 40%

**Figure 8:**
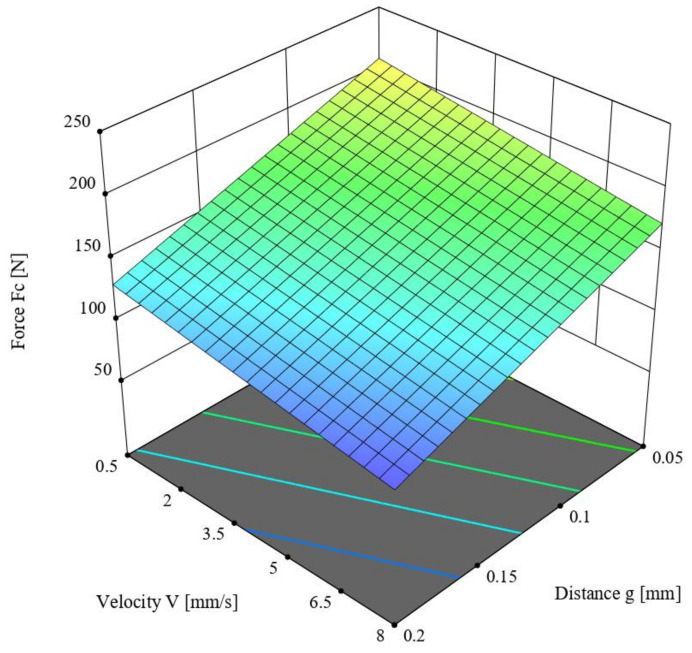
The variance of the cutting force *F_c_* as a function of offset *g* between the blade and the counter-blade and the linear velocity of the blade *V* for the straw stems with a moisture content of 40%.

This chapter describes the ANOVA variance analysis performed for the value of the cutting force *F_c_* (N) for the straw stem with a moisture content of 40%, depending on the offset between the blade and the counter-blade *g* (mm) as well as the knife blade velocity *V* (mm/s). Table 3 presents the obtained results. The linear model was employed in the analysis based on the criterion of statistical significance *p* = 0.1, for which R^2^ = 0.6995. According to Table 3, the F-value of the model was 13.97, which means the model was significant. Meanwhile, the *p*-value for individual model components was lower than 0.05, which confirms the significance of these components. The difference between the predicted R^2^ = 0.5542 and the adjusted R^2^ = 0.6494 coefficients of determination, which was lower than 0.2, along with the Adeq Precision value of 9.9093, indicates the usefulness of the model to estimate the value of the force. The obtained model is presented in Formula (2), and its graphical representation is shown in Figure 8.

*F_c_* = 236.507 − 519.071 × *g* − 4.68771 × *V*, (2)

Here, *F_c_* is the cutting force (N), *g* is the offset between the blade and the counter-blade (mm), and *V* is the knife blade velocity (mm/s).

In analyzing the parameter values in Table 3, the offset *g* had a significant influence (*p* < 0.001) on the value of the cutting force *F_c_*. A lower *F_c_* value was achieved for an offset *g* = 0.2 mm and velocity *V* = 8 mm/s.

**Figure 9:**
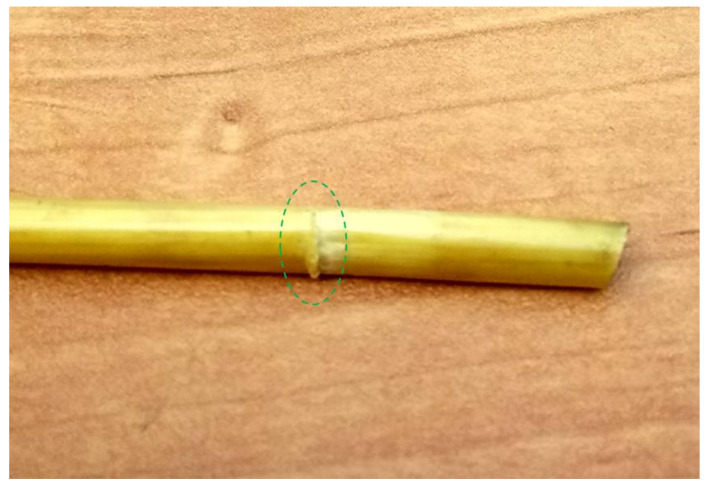
General view of a partially cut straw stem from a test attempt with an offset value *g* = 0.2 mm.

The experimental study of the cutting process carried out above, together with the ANOVA variance analysis of the obtained results concerning the cutting force value *F_c_* for both values of moisture content in straw stems, gives grounds for the following main conclusions:

- Higher values of the cutting force were recorded at the higher value (40%) of moisture content in the straw stems:
- Lower values of the cutting force *F_c_* in both cases of straw stem moisture contents were recorded for the parameter values *g* = 0.2 mm and *V* = 8 mm/s (see Figure 7 and Figure 8);
- In the course of the experiment, it was observed that for the offset *g* = 0.2 mm in selected tests, a partial separation of the stem occurred, resulting in a phenomenon of forcing it through the gap between the blade and the counter-blade (see Figure 9). This eliminated the offset value of *g* = 0.2 mm as a valid parameter (set-point) for use in the second stage of the experiment.

The first stage of the study, presented above, was carried out for a blade angle value *α =* 0° in order to determine which values of the *g*, *V*, and straw moisture content parameters led to the lowest value of the cutting force *F_c_*. This information was utilized in the second stage of the study.

### 3.2. Results of the Second Stage of the Study with Blade Angle α ≠ 0° and Rake Angle β ≠ 0°

This section discusses and analyzes the results of the experimental study carried out in the second part of the examination. Table 4 lists the maximum measured cutting force value *F_c_* for individual values of the blade angle *α* and the rake angle *β* for all test attempts. The test was repeated ten times for each value of both angles.

The results obtained were employed for the purpose of ANOVA variance analysis in order to identify the interactions among the input parameters (set-points) of the cutting process (see Figure 5) and the response value of the measured cutting force *F_c_*.

#### Multivariate Analysis of the Cutting Force F_c_ for Cutting Straw in the Second Stage of the Experiment

**Figure 10:**
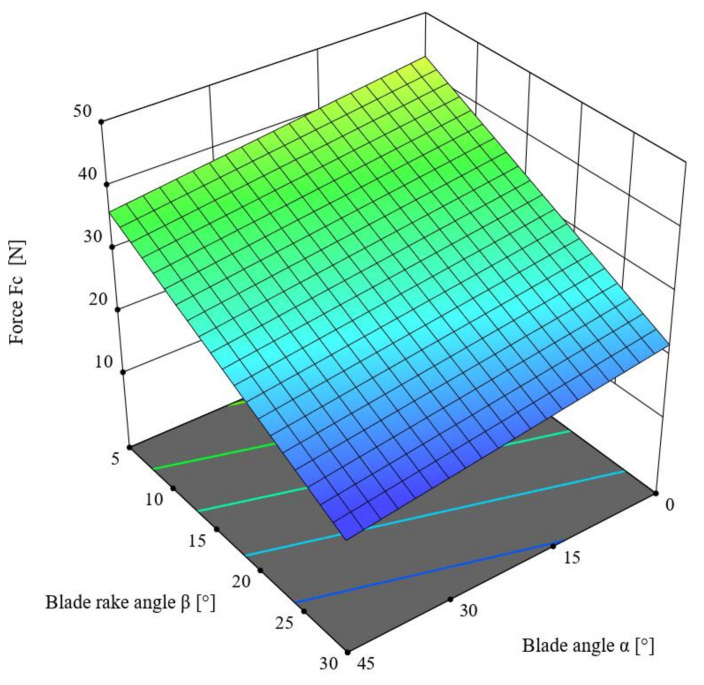
The variance of the cutting force *F_c_* as a function of the blade angle *α* and the rake angle *β* for offset value *g* = 0.1 mm and linear blade velocity of *V* = 8 mm/s.

This chapter describes the ANOVA variance analysis performed for the value of the cutting force *F_c_* (N) for the straw stem with a moisture content of 10%, depending on the offset between the blade and the counter-blade *g* = 0.1 (mm) as well as the knife blade velocity *V* = 8 (mm/s), depending on the value of the blade angle *α* and the rake *β*. Table 5 presents the obtained results. The linear model was employed in the analysis based on the criterion of statistical significance *p* = 0.1, for which R^2^ = 0.7721. According to Table 5, the F-value of the model was 15.25, which means that the model was significant. Meanwhile, the *p*-value for individual model components was lower than 0.05, which confirms the significance of these components. The difference between the predicted R^2^ = 0.5930 and the adjusted R^2^ = 0.7215 coefficients of determination, which was lower than 0.2, along with the Adeq Precision value of 9.8471, indicates the usefulness of the model to estimate the value of the force. The obtained model is presented in Formula (3), and its graphical representation is shown in Figure 10.

*F_c_* = 47.2387 − 0.150667 × *α* − 0.840921 × *β*, (3)

Here, *F_c_* is the cutting force (N), *α* is the blade angle (°), and *β* is the blade rake angle (°).

An analysis of the parameter values provided in Table 5 indicates that the value of the cutting force *F_c_* was significantly affected by the blade rake angle *β* (*p* < 0.001). The statistical F-value for this variable was F = 28.09, in comparison to the F-value of the blade angle *α*, which was F = 2.40. A further conclusion is that the lowest value of the cutting force *F_c_* necessary to separate the triticale straw was recorded for the parameter values *α* = 45° and *β* = 30°.

## 4. Discussion

**Figure 11:**
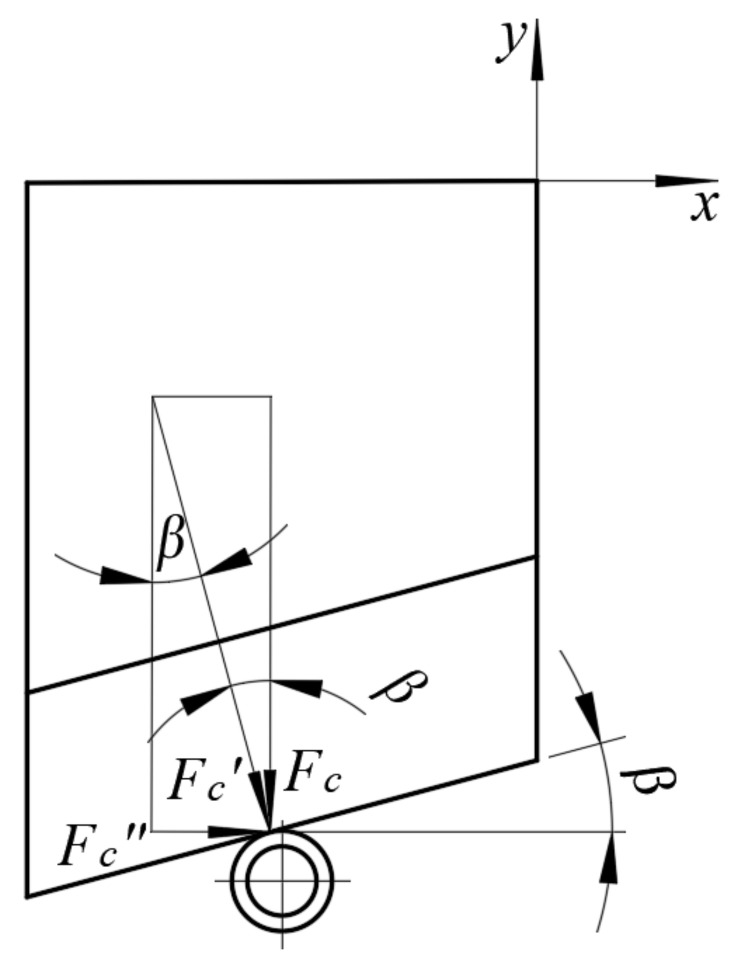
Analyzed force distribution on the *xy* plane.

The distribution of forces on the knife blade was analyzed considering two planes of the assumed Cartesian coordinate system *x*, *y*, *z*. Figure 11 presents the force distribution on the blade considering the analysis of the influence of the variance of the rake angle *β* along the *xy* plane.

Based on the analyzed force distribution along the *xy* plane, as in Figure 11, it was assumed that the force value *F_c_* recorded in the course of the experiment was the vertical component of the force *F_c_*′. The force *F_c_*′ may then be considered the main cutting force enacted perpendicular to the cutting edge of the knife. The second component of the cutting force *F_c_*′ was the horizontal component enacted along the direction of the axis *x*. The vectors of all the above-mentioned forces were applied at the point of contact of the cutting edge of the blade and the straw (see Figure 11). The dependence for calculating the *F_c_*′ can be expressed as below (4):

(4) $$F_{c}^{\prime }=\frac{F_{c}}{\cos\beta }$$

where

*F_c_*′—force perpendicular to the edge of the knife blade (N);
*F_c_*—measured cutting force (N);
*β*—blade rake angle (°).

The dependence on the horizontal component *F_c_*″ is expressed as below (5):

(5) $$F_{c}^{\prime \prime }=F_{c}\;\cdot \;\tan\beta$$

where

*F_c_*″—force horizontal to the measured *F_c_* (N);
*F_c_*—measured vertical component of the cutting force (N);
*β*—blade rake angle (°).

Therefore, the ratio of the horizontal component *F_c_*″ to the measured cutting force *F_c_* can be expressed as below (6):

(6) $$\frac{F_{c}^{\prime \prime }}{F_{c}}=\tan\beta$$

The value of the ratio expressed as (5) can be considered a coefficient [33,34,35,36,37,38] that determines the percentage share of the horizontal force component *F_c_*″ relative to the force value *F_c_* measured in the experiment. This can be interpreted in the following manner: a too-high ratio of the horizontal force component *F_c_*″ may contribute to the undesired phenomenon of the straw stem moving in the direction of the axis *x* (see Figure 11), consequently leading to the straw moving from under the blade, and thus preventing separation, as this partial displacement leads to a longer path to be traveled by the knife blade. This has a negative effect on the energy efficiency of the process, as it directly increases its duration. It would be, therefore, necessary to mount an additional component in the cutting station to eliminate the possibility of the movement of the straw stems, thus increasing the cost of the cutting station. Similar conclusions may be drawn for machinery that cuts a large amount of straw stems simultaneously.

Table 6 lists the variance of individual force values together with the *F_c_*″/*F_c_* ratio depending on the changes in the values of the angles *α* and *β*.

**Figure 12:**
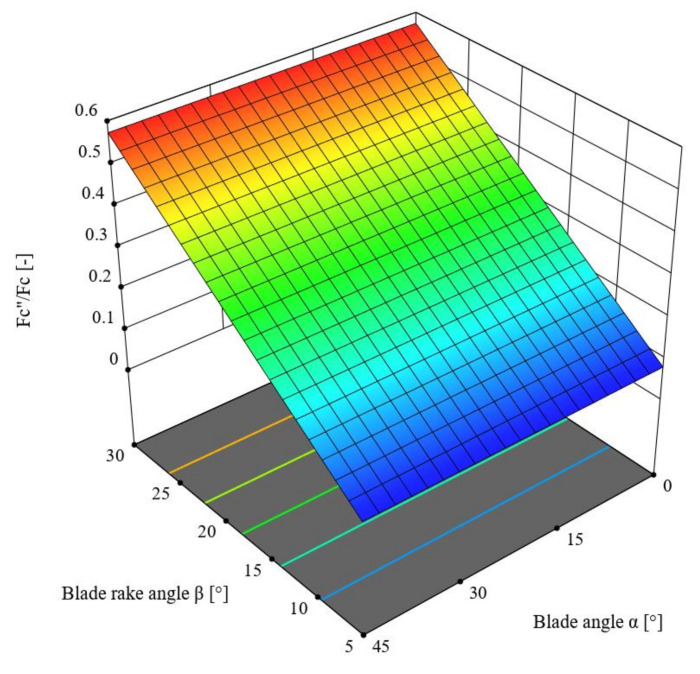
Variance of the *F_c_*″/*F_c_* force ratio as a function of the blade angle *α* and the rake angle *β*.

This chapter describes the ANOVA variance analysis performed for the *F_c_*″/*F_c_* ratio depending on the change in the values of the angles *α* and *β.* Table 7 presents the obtained results. The reduced linear model was employed in the analysis based on the criterion of statistical significance *p* = 0.1, for which R^2^ = 0.9987. According to Table 7, the F-value of the model was 7772.22, which means the model was significant. Meanwhile, the *p*-value for the blade rake angle *β* was lower than 0.001, which confirms the significance of this component. The difference between the predicted R^2^ = 0.9983 and the adjusted R^2^ = 0.9986 coefficients of determination, which was lower than 0.2, along with the Adeq Precision value of 151.6901, indicates the usefulness of the model to estimate the value of the force. The obtained model is presented in Formula (7), and its graphical representation is shown in Figure 12.

*F_c_*″/*F_c_* = −0.0170033 + 0.019676 × *β,* (7)

Here, *F_c_* is the cutting force (N), *F_c_*″ is the horizontal force component by Figure 11 (N), and *β* is the blade rake angle (°)

It follows from the analysis of the parameters in Table 7 that the only significant influence (which is why Table 7 only includes the rake angle *β*, and the lines of the surface graph are parallel to the axis of variance of the blade angle *α*, which further confirms that the variance of this angle value is not significant) on the value of the *F_c_*″/*F_c_* ratio is that of the blade rake angle *β* (*p* < 0.001), where the statistical F-value for this variable is F = 7772.22. The lowest value of the *F_c_*″/*F_c_* ratio was recorded for the entire range of values of the blade angle *α* and the angle *β* = 5°.

**Figure 13:**
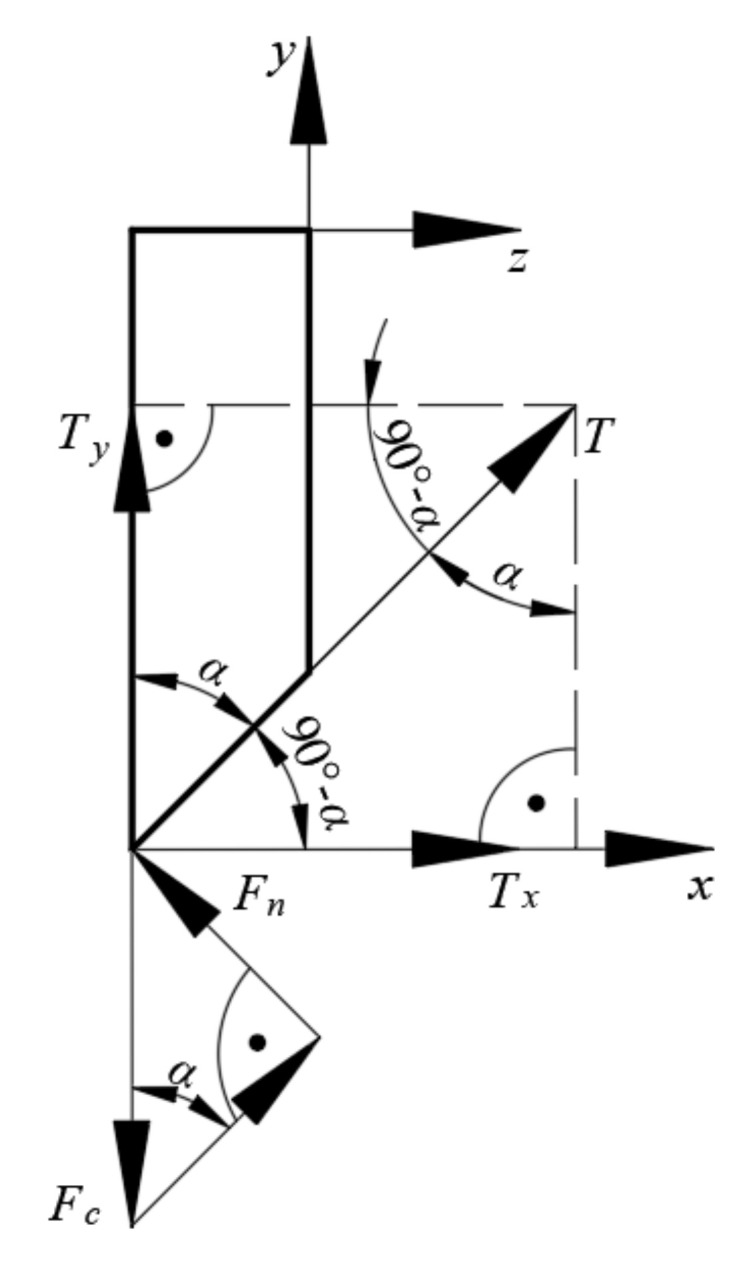
Analyzed distribution of forces on the knife blade considered along the *yz* plane.

In the next step, the distribution of forces on the knife blade along the *yz* plane was analyzed, as provided in Figure 13. This led to determining the force ratio *F_w_*/*F_c_*, which serves as an indicator of the efficiency of the cutting process, with the highest possible values thereof being desirable. The force component *F_w_* is explained in greater detail below.

In analyzing the array of forces projected on the axis *y* (see Figure 13), the following dependence (8) can be obtained, indicating that the force *F_c_* is a sum of the working force *F_w_* and the vertical component *T_y_* of the frictional force *T*:

(8) $$F_{c}=F_{w}+T_{y}$$

where

*F_c_*—force measured in the course of the cutting process of triticale straw (N);
*F_w_*—working force (N);
*T_y_*—vertical component of the frictional force *T* (N).

Meanwhile, the value of the component of the working force can be expressed as follows:

(9) $$F_{w}=F_{s}+F_{m}$$

where

*F_w_*—working force (N);
*F_s_*—force necessary to only cut (separate) the straw material (N);
*F_m_*—force necessary to overcome the resistances of the cut material; in other words, it is the force necessary to overcome the reaction force of the material compressed by the knife blade (N).

The formula for the vertical component *T_y_* of the frictional force *T* can be expressed as follows (see Figure 13):

(10) $$T_{y}=T\;\cdot \cos\alpha =\;\mu \cdot F_{n}\cdot \cos\alpha =\mu \cdot F_{c}\cdot \cos\;\cdot \left(90{}^{\circ}-\alpha \right)\;\cdot \cos\;\alpha \;$$

where (see Figure 13)

*T*—frictional force (N);
*T_y_*—vertical component of the frictional force *T* (N);
*F_n_*—normal force of the frictional force *T* (N);
*F_c_*—force registered during the cutting process of triticale straw (N);
*α*—knife blade angle (°);
*μ*—coefficient of friction; according to Richter (1954), *μ* = 0.3 [39].

Based on the above dependences, a formula was developed to determine the percentage share of the force *F_w_* in the force *F_c_*:

(11) $$F_{c}=F_{w}+T_{y},$$

(12) $$F_{c}=F_{w}+\mu \cdot F_{c}\cdot \cos\;\cdot \left(90{}^{\circ}-\alpha \right)\;\cdot \cos\;\alpha ,$$

(13) $$F_{c}-\mu \cdot F_{c}\cdot \cos\;\cdot \left(90{}^{\circ}-\alpha \right)\;\cdot \cos\;\alpha =F_{w},$$

(14) $$F_{c}\cdot \left(1-\mu \cdot \cos\;\cdot \left(90{}^{\circ}-\alpha \right)\;\cdot \cos\;\alpha \right)=F_{w},$$

Finally,

(15) $$\;\frac{F_{w}}{F_{c}}=\left(1-\mu \cdot \cos\;\cdot \left(90{}^{\circ}-\alpha \right)\;\cdot \cos\;\alpha \right).$$

Based on the above dependence, the value of the ratio *F_w_*/*F_c_* can be calculated depending on the value of the blade angle *α* and the rake angle *β*. As mentioned previously, this ratio can be defined as the efficiency of the cutting process, with the input parameters leading to the highest possible value of this parameter. This means that the recorded force value *F_c_* was utilized to the highest possible degree to separate the material and to the lowest possible degree to overcome resistances related to friction (see Equation (11)).

**Figure 14:**
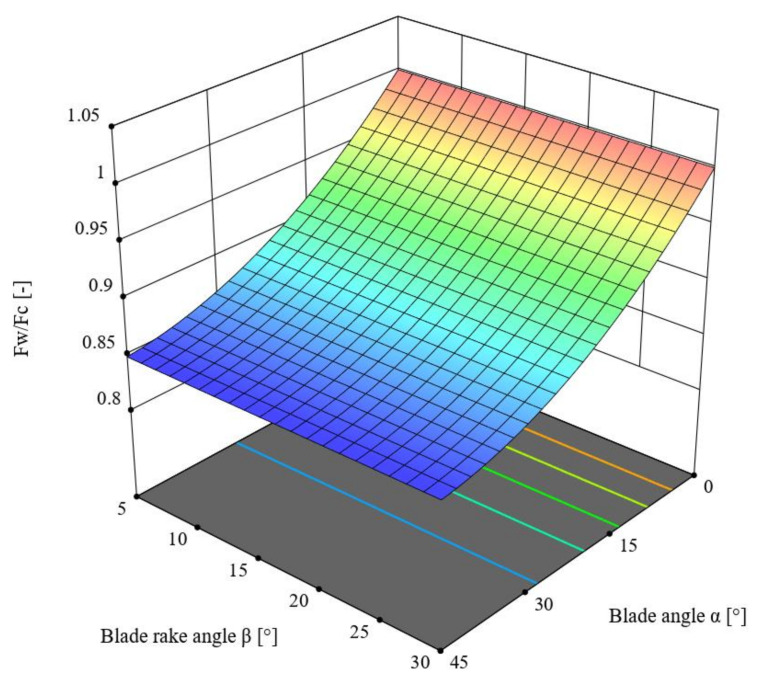
Variance of the force ratio *F_w_*/*F_c_* as a function of the blade angle *α* and the blade rake angle *β*.

This chapter describes the ANOVA variance analysis performed for the *F_w_*/*F_c_* ratio depending on the change in the values of the angles *α* and *β.* Table 8 presents the obtained results. The reduced quadratic model was employed in the analysis based on the criterion of statistical significance *p* = 0.1, for which R^2^ = 0.9992. According to Table 8, the F-value of the model was 5613.15, which means the model was significant. Meanwhile, the *p*-value for the knife blade angle *α* and its value *α^2^* was lower than 0.001, which confirms the significance of these components. The difference between the predicted R^2^ = 0.9987 and the adjusted R^2^ = 0.9990 coefficients of determination, which was lower than 0.2, along with the Adeq Precision value of 159.4964, indicates the usefulness of the model to estimate the value of the force. The obtained model is presented in Formula (16), and its graphical representation is shown in Figure 14.

*F_w_*/*F_c_* = 1.00073 − 0.00611115 × *α* + 0.000061 × *α^2^,* (16)

Here, *F_c_* is the cutting force (N), *F_w_* is the work force (N), *α* is the blade angle (°), and *β* is the blade rake angle (°).

The analysis of the parameter values provided in Table 8 indicates that the value of the ratio *F_w_*/*F_c_* was significantly influenced by the blade angle *α* (*p* < 0.001), and the F-value for this variable was F = 10599.62. The lowest value of the *F_w_*/*F_c_* ratio was recorded for the angle value *α* = 45° as well as the entire range of values of the angle *β*.

### Optimization of the Selection of Cutting Process Parameters

Based on the analyses of the force distribution along the planes *xy* and *yz* and the determined values of the ratios *F_c_″*/*F_c_* and *F_w_*/*F_c_*, an optimization was carried out [27,40] with the view of seeking the optimum values of the input parameters of the cutting process based on the optimization criteria provided in Table 9.

The results of the optimization are provided in Table 10 as a function of the variable weight value for the force *F_c_* and the criterion for the ratios *F_c_″*/*F_c_* and *F_w_*/*F_c_*. The weighing was modified by increasing its value for the value of *F_c_* at the same time as reducing, by the same amount, the weighing value for the remaining criteria, i.e., the above-mentioned force ratios, whereas the increase in weight for the force value *F_c_* stands for a higher prioritization for minimizing the cutting force.

The table above presents the results determined from the optimization according to the criteria set in Table 9. The listed parameter values were determined on the basis of a prior ANOVA variance analysis and the obtained models of interaction for the individual input and output (response) parameters of the analyzed triticale straw-cutting process. This leads to the conclusion that the optimal blade angle value *α* was close to zero. In this case, considering the weight value of 0.8, the force value *F_c_* achieved the minimum value, thereby minimizing the energy necessary to separate the straw stem, considering a blade angle value *α* close to zero and a rake angle of *β* = 26°. However, this comes at a trade-off, necessitating a higher share of the horizontal force component *F_c_″* as follows from the ratio: *F_c_″*/*F_c_* = 0.49. The efficiency of the entire range of values for the optimization weights remained the same and was close to 100%. When reducing the weight value for the criterion of minimizing the cutting force value *F_c_* while simultaneously increasing the value for both ratios *F_c_″*/*F_c_* and *F_w_*/*F_c_*, the value of the cutting force increased the *F_c_*. The value of the blade angle *α* was close to zero for the entire range of values of the optimization weights. On the other hand, the value of the rake angle *β* decreased (see Table 10). Considering it desirable to maintain the lowest possible cutting force value at the same time as minimizing the horizontal component *F_c_″*, the weights can be set as indicated in the final row of Table 10. In this case, the high process efficiency was maintained since *F_w_*/*F_c_* = 0.992, preserving the low value of the horizontal force component *F_c_*″ based on the value of the ratio *F_c_″*/*F_c_* = 0.2. Unfortunately, the cutting force *F_c_* increased by over 50% relative to its minimum value, which was achieved for the respective weight values of 0.8, 0.1, and 0.1, as provided in Table 10. 

## 5. Conclusions

Based on the results obtained in the course of the experimental study and performed analyses and optimizations, one may draw the following conclusions:

- The ANOVA variance analysis of the obtained study results in the first stage performed on the set parameters of the triticale straw-cutting process, i.e., blade angle value *α* = 0°, rake angle *β* = 0°, offset *g* = 0.05, 0.1, and 0.2 mm, and linear velocity *V* = 0.5, 1, 2, 4, and 8 mm/s indicates that the lowest cutting force value *F_c_* was achievable for the parameter values *g* = 0.2 mm and *V* = 8 mm/s. However, with the value *g* = 0.2 mm, there were incidents of incorrect cutting of the triticale straw, which eliminated the possibility of using this set-point value. Therefore, in the second stage of the examination, the values *g* = 0.1 mm and blade velocity *V* = 8 mm/s were used.
- The ANOVA variance analysis of the obtained experimental results carried out in the second stage of the testing indicates that the lowest *F_c_* value necessary to cut the triticale straw was achievable with *α* = 45° and *β* = 30°, together with the parameter values assumed in the first stage of the examination, i.e., offset *g* = 0.1 mm and blade velocity *V* = 8 mm/s.
- Considering the analysis of the force distribution on the knife blade (Section 4), which led to determining the force ratios *F_c_″*/*F_c_* and *F_w_*/*F_c_*, and based on the performed optimization following the assumed criteria (see Section Optimization of the Selection of Cutting Process Parameters), it was possible to determine the optimum blade angle *α* (assuming the values: *g* = 0.1 mm and *V* = 8 mm/s), which was equal to *α* ≅ 0°, and the blade rake angle *β* was in the value range of 5–26°. The selection of a specific value from the indicated range depends on the weighting criterion adopted in the optimization process. These values can be decided at the discretion of the cutting machine constructor.

## Figures and Tables

**Table 1:** List of average measured values of the cutting force *F_c_* from all the tests in the course of the experiment.

*g* (mm)	*V* (mm/s)	*F_c_* (N) 10%	*F_c_* (N) 40%
0.05	0.5	75.1	211.4
0.05	1	66.8	153.3
0.05	2	84.2	242.3
0.05	4	91.6	214.5
0.05	8	74.7	158.1
0.1	0.5	100.4	198.3
0.1	1	82.1	166.7
0.1	2	117.1	196
0.1	4	68.4	154.4
0.1	8	53.7	135.9
0.2	0.5	61	123.7
0.2	1	62.3	128.3
0.2	2	80.4	97
0.2	4	47.1	140.5
0.2	8	53.9	101

**Table 2:** ANOVA results. Dependent variable—cutting force *F_c_* (N).

Source	Sum of Squares	df ^a^	Mean Square	F-Value	*p*-Value	
Model	1790.3	2	895.2	33.5	0.00700	Significant
*g*	1041.4	1	1041.4	38.9	0.00719	
*V*	749.0	1	749.0	28.0	0.01201	

^a^ Degrees of freedom.

**Table 3:** ANOVA results. Dependent variable—cutting force *F_c_* (N).

Source	Sum of Squares	df ^a^	Mean Square	F-Value	*p*-Value	
Model	18169.4	2	9084.7	14.0	0.0007	Significant
*g*	15717.0	1	15717.0	24.2	0.0004	
*V*	2452.4	1	2452.4	3.8	0.0760	

^a^ degrees of freedom.

**Table 4:** Breakdown of the cutting force values *F_c_* from each of the ten tests, together with an average value for each angle *α* and *β* value.

		*F_c_* (N)										
No.	*α* = 0°, *β* = 5°	*α* = 15° *β* = 5°	*α* = 30°, *β* = 5°	*α* = 45°, *β* = 5°	*α* = 0°, *β* = 15°	*α* = 15°, *β* = 15°	*α* = 30°, *β* = 15°	*α* = 45°, *β* = 15°	*α* = 0°, *β* = 30°	*α* = 15°, *β* = 30°	*α* = 30°, *β* = 30°	*α* = 45°, *β* = 30°
1.	44.7	50.5	42.1	34.9	28.9	36.1	23.1	23.7	17.8	25.3	22.2	18.7
2.	42.4	52.3	37.1	28.7	30.3	32.8	28.0	24.0	17.1	25.0	17.3	17.9
3.	31.5	58.2	36.2	28.9	27.7	34.2	25.4	23.2	19.0	25.3	16.7	16.3
4.	38.5	61.6	39.9	31.0	30.2	34.9	32.7	25.6	17.3	23.1	17.5	15.6
5.	36.3	46.4	42.7	35.8	34.2	31.9	26.6	26.6	16.9	25.1	17.7	15.3
6.	46.3	60.0	38.0	34.8	32.5	30.9	29.2	25.1	16.6	25.3	17.2	15.8
7.	36.5	64.9	35.7	31.8	32.6	33.2	26.9	27.3	17.2	23.4	22.6	15.8
8.	38.1	47.9	34.5	27.6	33.7	36.9	29.7	27.2	17.6	25.1	17.5	17.3
9.	31.3	45.1	41.0	34.3	34.8	31.4	31.0	27.4	17.4	24.3	18.1	17.2
10.	36.6	53.6	33.5	29.5	33.3	33.9	26.4	25.9	16.7	23.0	16.5	16.7
*F_cavg_* (N)	38.2	54.1	38.1	31.7	31.8	33.6	27.9	25.6	17.4	24.5	18.3	16.7

**Table 5:** ANOVA results. Dependent variable—cutting force *F_c_* (N).

Source	Sum of Squares	df ^a^	Mean Square	F-Value	*p*-Value	
Model	972.3	2	486.2	15.3	0.0013	Significant
*α*	76.6	1	76.6	2.4	0.1555	
*β*	895.7	1	895.7	28.1	0.0005	

^a^ degrees of freedom.

**Table 6:** Breakdown of the force values *F_c_*, *F_c_*′, and *F_c_*″ together with the *F_c_*″/*F_c_* ratio as a function of the variance of the angles *α* and *β*.

*α* (°)	*β* (°)	*F_c_* (N)	*F_c_′* (N)	*F_c_*″ (N)	*F_c_*″/*F_c_* (-)
0	5	38.2	38.4	3.3	0.087
0	15	31.8	32.9	8.5	0.268
0	30	17.4	20.1	10.1	0.577
15	5	54.1	54.3	4.7	0.087
15	15	33.6	34.8	9.0	0.268
15	30	24.5	28.3	14.1	0.577
30	5	38.1	38.3	3.3	0.087
30	15	27.9	28.9	7.5	0.268
30	30	18.3	21.1	10.6	0.577
45	5	31.8	31.9	2.8	0.087
45	15	25.6	26.5	6.9	0.268
45	30	16.7	19.3	9.6	0.577

**Table 7:** ANOVA results. Dependent variable—value quotient *F_c_*″/*F_c_*(N).

Source	Sum of Squares	df ^a^	Mean Square	F-Value	*p*-Value	
Model	0.4904	1	0.4904	7772.2	<0.0001	Significant
*β*	0.4904	1	0.4904	7772.2	<0.0001	

^a^ degrees of freedom.

**Table 8:** ANOVA results. Dependent variable—value quotient—*F_w_*/*F_c_*(N).

Source	Sum of Squares	df ^a^	Mean Square	F-Value	*p*-Value	
Model	0.0405	2	0.0202	5613.2	<0.0001	Significant
*α*	0.0382	1	0.0382	10599.6	<0.0001	
*α* ^2^	0.0023	1	0.0023	626.7	<0.0001	

^a^ Degrees of freedom.

**Table 9:** Assumed optimization criteria.

Input/Output Variable	Goal	Lower Limit	Upper Limit
*α* (°)	is in range	0	45
*β* (°)	is in range	5	30
*F_c_* (N)	minimize	16.7	54.1
*F_c_″*/*F_c_* (-)	minimize	0.0875	0.577
*F_w_*/*F_c_* (-)	maximize	0.85	0.99

**Table 10:** Process parameters meeting the optimization criteria.

Weight *F_c_*/(*F_c_*_″_/*F*_c_)/(*F_w_*/*F*_c_)	Blade Angle *α* (°)	Blade Rake Angle *β* (°)	*F_c_* (N)	*F_c_*_″_/*F*_c_ (-)	*F_w_*/*F*_c_ (-)
0.1/0.45/0.45	0.12	5.3	42.8	0.087	0.999
0.2/0.4/0.4	0.12	5.3	42.8	0.087	0.999
0.3/0.35/0.35	0.12	9.5	39.2	0.17	0.999
0.4/0.3/0.3	0.12	13.8	35.7	0.25	0.999
0.5/0.25/0.25	0.12	17.4	32.6	0.33	0.999
0.6/0.2/0.2	0.12	20.6	29.9	0.39	0.999
0.7/0.15/0.15	0.12	23.4	27.5	0.44	0.999
0.8/0.1/0.1	1.4	25.9	25.2	0.49	0.992
0.45/0.45/0.1	1.45	10.9	37.9	0.2	0.992

## Data Availability

The data presented in this study are available upon request from the corresponding author.

## References

[1] Larkin S., Ramage J., Scurlock J., Boyle G. (2004). Bioenergy. Renewable Energy: Power for a Sustainable Future.

[2] Chico-Santamarta L., Humphroes A., White D., Chaney K., Godwin R.J. Effect of Pre- and Post-Pelletization Storage of Canola (Oilseed Rape) Straw on the Quality and Properties of Pellets. Proceedings of the ASABE Annual International Meeting, Grand Sierra Resort and Casino.

[3] Liu R., Yu H., Huang Y. (2005). Structure and Morphology of Cellulose in Wheat Straw. Cellulose.

[4] Siwale W., Frodeson S., Berghel J., Henriksson G., Finell M., Arshadi M., Jonsson C. (2022). Influence on off-gassing during storage of Scots pine wood pellets produced from sawdust with different extractive contents. Biomass Bioenergy.

[5] Tanger P., Field J.L., Jahn C.E., DeFoort M.W., Leach J.E. (2013). Biomass for thermochemical conversion: Targets and challenges. Front. Plant Sci..

[6] Ben-Iwo J., Manovic V., Longhurst P. (2016). Biomass resources and biofuels potential for the production of transportation fuels in Nigeria. Renew. Sustain. Energy Rev..

[7] Tabil L., Adapa P., Kashaninejad M., Dos Santos Bernardes M.A. (2011). Biomass Feedstock Pre-Processing—Part 1: Pre-Treatment. Biofuel’s Engineering Process Technology.

[8] Orisaleye J.I., Jekayinfa S.O., Adebayo A.O., Ahmed N.A., Pecenka R. (2018). Effect of densification variables on density of corn cob briquettes produced using a uniaxial compaction biomass briquetting press. Energy Sources, Part A: Recover. Util. Environ. Eff..

[9] DaSilva D.A., Alesi L.S., Da Róz A.L., Santos L., Quadros T.M.C., Yamaji F.M. (2018). Effect of the Particle Size on Compaction of Elephant Grass Biomass. Rev. Virtual Quim..

[10] Wu P., Ma Y., Chen Y., Zhang Y., Wang H. (2014). Vibration-assisted Compaction of Biomass. Bioresources.

[11] Talaśka K., Malujda I., Wilczyński D. (2016). Agglomeration of Natural Fibrous Materials in Perpetual Screw Technique—A Challenge for Designer. Procedia Eng..

[12] Wilczyński D., Berdychowski M., Talaśka K., Wojtkowiak D. (2021). Experimental and numerical analysis of the effect of compaction conditions on briquette properties. Fuel.

[13] Bembenek M., Zięba A., Kopyściański M., Krawczyk J. (2020). Analysis of the Impact of the Consolidated Material on the Morphology of Briquettes Produced in a Roller Press. J. Mater. Eng. Perform..

[14] Bembenek M., Wdaniec P. (2019). Effect of crusher type and its parameters on the dry granulation of powders. Przemysł Chem..

[15] Bembenek M., Buczak M., Baiul K. (2022). Modelling of the Fine-Grained Materials Briquetting Process in a Roller Press with the Discrete Element Method. Materials.

[16] Whittaker C., Shield I. (2017). Factors affecting wood, energy grass and straw pellet durability—A review. Renew. Sustain. Energy Rev..

[17] Relova I., Vignote S., León M., Ambrosio Y. (2009). Optimisation of the manufacturing variables of sawdust pellets from the bark of Pinus caribaea Morelet: Particle size, moisture and pressure. Biomass Bioenergy.

[18] Castellano J.M., Gomez M., Fernández M., Esteban L.S., Carrasco J.E. (2015). Study on the effects of raw materials composition and pelletization conditions on the quality and properties of pellets obtained from different woody and non woody biomasses. Fuel.

[19] Bergman R., Cai Z., Carll C.G. (2010). Wood Handbook: Wood as an Engineering Material (Centennial Edition).

[20] Kruszelnicka W., Marczuk A., Kasner R., Bałdowska-Witos P., Piotrowska K., Flizikowski J., Tomporowski A. (2020). Mechanical and Processing Properties of Rice Grains. Sustainability.

[21] Kruszelnicka W., Opielak M., Ambrose K., Pukalskas S., Tomporowski A., Walichnowska P. (2022). Energy-Dependent Particle Size Distribution Models for Multi-Disc Mill. Materials.

[22] Bitra V.S., Alvin R., Womac A.R., Igathinathane C., Miu P.I., Yang Y.T., Smith D.R., Chevanan N., Sokhansanj S. (2009). Direct measures of mechanical energy for knife mill size reduction of switchgrass, wheat straw, and corn stover. Bioresour. Technol..

[23] Zastempowski M., Bochat A. (2020). Research Issues in the Process of Cutting Straw into Pieces. Sustainability.

[24] Barakat A., Monlau F., Solhy A., Carrere H. (2015). Mechanical dissociation and fragmentation of lignocellulosic biomass: Effect of initial moisture, biochemical and structural proprieties on energy requirement. Appl. Energy.

[25] Azadbakht M., Esmaeilzadeh E., Esmaeili-Shayan M. (2015). Energy consumption during impact cutting of canola stalk as a function of moisture content and cutting height. J. Saudi Soc. Agric. Sci..

[26] Wilczyński D., Talaśka K., Malujda I., Jankowiak P. (2018). Experimental research on biomass cutting process. MATEC Web Conf..

[27] Gao Y., Hu X., Tong S., Kan J., Wang Y., Kang F. (2022). Multi-objective optimization of peak cutting force and cutting energy consumption in cutting of Caragana korshinskii branches. Bioresources.

[28] Zhang C., Chen L., Xia J., Zhang J. (2019). Effects of blade sliding cutting angle and stem level on cutting energy of rice stems. Int. J. Agric. Biol. Eng..

[29] Song S., Zhou H., Xu L., Jia Z., Hu G. (2022). Cutting mechanical properties of sisal leaves under rotary impact cutting. Ind. Crops Prod..

[30] Vu V.-D., Nguyen T.-T., Chu N.-H., Ngo Q.-H., Ho K.-T., Nguyen V.-D. (2020). Multiresponse Optimization of Cutting Force and Cutting Power in Chopping Agricultural Residues Using Grey-Based Taguchi Method. Agriculture.

[31] Mathanker S.K., Grift T.E., Hansen A.C. (2015). Effect of blade oblique angle and cutting speed on cutting energy for energycane stems. Biosyst. Eng..

[32] Johnson P.C., Clementson C.L., Mathanker S., Grift T.E., Hansen A.C. (2012). Cutting energy characteristics of Miscanthus x giganteus stems with varying oblique angle and cutting speed. Biosyst. Eng..

[33] Ventura C.E.H., Hassui A. (2013). Evaluation of static cutting forces and tool wear in HSM process applied to pocket milling. Int. J. Adv. Manuf. Technol..

[34] Voss B.M., Pereira M.P., Rolfe B.F., Doolan M.C. (2017). Using stamping punch force variation for the identification of changes in lubrication and wear mechanism. J. Phys. Conf. Ser..

[35] Choudhury S., Kishore K. (2000). Tool wear measurement in turning using force ratio. Int. J. Mach. Tools Manuf..

[36] Akyürek F., Yaman K., Tekiner Z. (2017). An Experimental Work on Tool Wear Affected by Die Clearance and Punch Hardness. Arab. J. Sci. Eng..

[37] Gouarir A., Martínez-Arellano G., Terrazas G., Benardos P., Ratchev S. (2018). In-process Tool Wear Prediction System Based on Machine Learning Techniques and Force Analysis. Procedia CIRP.

[38] Siddhpura A., Paurobally R. (2012). A study of the effects of friction on flank wear and the role of friction in tool wear monitoring. Aust. J. Mech. Eng..

[39] Richter D. (1954). Friction coefficients of some agricultural materials. Agric. Eng..

[40] Wojtkowiak D., Talaśka K. (2019). Determination of the effective geometrical features of the piercing punch for polymer composite belts. Int. J. Adv. Manuf. Technol..

